# The Effect of Blood Rheology and Inlet Boundary Conditions on Realistic Abdominal Aortic Aneurysms under Pulsatile Flow Conditions

**DOI:** 10.3390/bioengineering10020272

**Published:** 2023-02-20

**Authors:** Konstantinos Tzirakis, Yiannis Kamarianakis, Nikolaos Kontopodis, Christos V. Ioannou

**Affiliations:** 1 Department of Mechanical Engineering, Hellenic Mediterranean University, 71410 Heraklion, Crete, Greece; 2 Data Science Group, Institute of Applied and Computational Mathematics, Foundation for Research & Technology-Hellas, 70013 Heraklion, Crete, Greece; 3 Vascular Surgery Department, Medical School, University of Crete, 71003 Heraklion, Crete, Greece

**Keywords:** abdominal aortic aneurysm, inlet velocity boundary condition, blood rheological models, finite volume method, robust two-way analysis of variance, post hoc tests

## Abstract

Background: The effects of non-Newtonian rheology and boundary conditions on various pathophysiologies have been studied quite extensively in the literature. The majority of results present qualitative and/or quantitative conclusions that are not thoroughly assessed from a statistical perspective. Methods: The finite volume method was employed for the numerical simulation of seven patient-specific abdominal aortic aneurysms. For each case, five rheological models and three inlet velocity boundary conditions were considered. Outlier- and heteroscedasticity-robust ANOVA tests assessed the simultaneous effect of rheological specifications and boundary conditions on fourteen variables that capture important characteristics of vascular flows. Results: The selection of inlet velocity profiles appears as a more critical factor relative to rheological specifications, especially regarding differences in the oscillatory characteristics of computed flows. Response variables that relate to the average tangential force on the wall over the entire cycle do not differ significantly across alternative factor levels, as long as one focuses on non-Newtonian specifications. Conclusions: The two factors, namely blood rheological models and inlet velocity boundary condition, exert additive effects on variables that characterize vascular flows, with negligible interaction effects. Regarding thrombus-prone conditions, the Plug inlet profile offers an advantageous hemodynamic configuration with respect to the other two profiles.

## 1. Introduction

Cardiovascular diseases (CVDs) are the leading cause of death globally [1]. An estimated 17.9 million individuals died from CVDs in 2019, representing more than 30% of all deaths worldwide. They comprise a group of disorders that affect the heart and blood vessels such as rheumatic and congenital heart disease, cerebrovascular disease (stroke), aortic aneurysms, and pulmonary embolism. Specifically, abdominal aortic aneurysms (AAAs) represent the 13th leading cause of death in Western societies [2]. It is typically defined as an at least 1.5-fold increase in vessel diameter and its rupture constitutes the most dangerous implication, which is usually accompanied by a catastrophic insult with an overall mortality between 70% and 90% [3,4]. The traditional criterion for rupture risk assessment is the AAA’s maximum diameter that should not exceed the threshold of 55 mm. Even though this criterion has served the medical community over the years, it is reasonable to question the concept that a single parameter can sufficiently fulfill the needs of all patients. To this end, additional procedures have been developed towards increasing rupture risk assessment, such as surgery outcome prediction [5,6], noninvasive imaging [7,8], and numerical simulations through computational fluid dynamics (CFD) [9,10,11,12].

The latter approach provides an advantage to researchers and clinicians, since it can resolve spatial and temporal resolution of blood flow in high detail without the need of in vivo measurements, which are invasive and possibly demanding to perform. A prerequisite, though, of almost any CFD simulation is an appropriate set of conditions that formulate a well-defined problem to be solved. These conditions depend on the question under consideration, but typically involve assumptions on blood flow rheology, inlet/outlet boundary or initial conditions, and biomechanical properties of relevant vessels amongst others. To this end, numerous works have appeared in the literature studying the effects of various assumptions on the hemodynamic behavior of idealized or realistic geometries.

Neofytou et al. [13] studied the effects of different blood rheological models on a stenosis and an abdominal aortic aneurysm, focusing on the distribution of wall shear stress in the vicinity of vessel abnormalities. Arzani [14] proposed a residence time non-Newtonian model that accounts for the rouleaux formation time-scale in blood shear-thinning behavior, while Bilgi et al. [15] concluded that a Carreau fluid through a hyperelastic vessel behaves substantially different than a Newtonian fluid with a linearly elastic arterial wall. Skiadopoulos et al. [16] compared three blood flow models in patient-specific cardiovascular systems and concluded that the Newtonian assumption is valid only for high shear and flow rates.

The impact of boundary conditions has also been examined quite extensively. Morbiducci et al. [17] studied idealized versus measured velocity profiles as inlet boundary conditions in the human aorta. Similarly, Youssefi et al. [18] performed a numerical study on the effect of various inlet boundary conditions in the thoracic aorta and concluded that idealized velocity profiles can potentially lead to significant alterations of velocity patterns and magnitudes in the aorta. More studies on aortic flows include the works of Madhavan et al. [19] and Fuchs et al. [20], where it was shown that simulation results were in general sensitive to the choice of boundary conditions. The impact of inlet boundary conditions on blood flow has also been examined in various other physiologies such as stented coronary arteries [21], carotid bifurcations [22], intracranial aneurysms [23], and abdominal aortic aneurysms [24].

The plethora of available studies in the literature present a qualitative and/or quantitative evaluation of the effects of rheological models and boundary conditions on the hemodynamic behavior of various pathophysiologies, without usually assessing observed differences from a statistical perspective. The aim of this work is to analyze the effects of blood flow models and inlet boundary conditions on patient-specific abdominal aortic aneurysms, with statistical tools which are robust to deviations from the standard assumptions of conventional analysis of variance (ANOVA): equal levels of uncertainty across groups (homoscedasticity) and absence of outliers. Specifically, seven AAA cases were considered, with five different models and three inlet boundary conditions, yielding 105 numerical simulations in total. Fourteen response variables that characterize vascular flows were computed per simulation and analyzed with modern statistical methods. The response variables quantify (a) the average tangential force on the wall over the entire cycle; (b) the oscillatory nature of the flow; (c) flow asymmetry; (d) flow dispersion; and (e) the extent of thrombogenic stimulating environments.

The manuscript is organized as follows: Section 2 reports the segmentation/reconstruction and meshing process for the seven cases considered. It also formulates the mathematical framework that underlies numerical simulations and presents the adopted velocity inlet boundary conditions and the alternative rheological specifications. Furthermore, it defines the hemodynamic variables, as well as the robust statistical models and hypothesis tests employed for their analysis. Section 3 presents a mesh convergence study and reports the outcomes of the statistical analysis, which evaluates the effect of inlet velocity and blood flow model on the overall hemodynamic behavior of the AAAs under consideration. Finally, Section 4 presents a thorough discussion on our findings and Section 5 concludes the paper.

## 2. Materials and Methods

### 2.1. Image Segmentation, Surface Reconstruction, and Meshing

Seven AAA patients (denoted 2B, 7A, 14B, 16A, 31A, 41B, and 63A) underwent electrocardiogram-gated (ECG-gated) computed tomography scans to image the AAA geometry. Following [25], a Somatom definition flash, dual-source–dual-energy CT scanner (Siemens, Munich, Germany), with before and after contrast media administration with retrospective ECG-gated spiral acquisition, was used for imaging. A total effective dose of 5.5 mSv at 80 bpm non-ionic contrast was used with a slice thickness of 0.625 mm and image matrix size 512 × 512. The temporal resolution was set equal to 83 ms with 0.33 mm in-plane spatial resolution. Segmentation and surface reconstruction were performed manually using the open source software ITK_SNAP [26]. The generated surfaces were smoothed using VMTK [27]; specifically, the Taubin algorithm that preserves the volume under consideration [28] was implemented. A passband of 0.01 and 100 iterations were sufficient in all cases in order to remove the surface noise, without changing the AAA sac volume more than 0.15%. Finally, cylindrical extensions were added at the aorta and iliac arteries to allow flow development.

**Figure 1:**
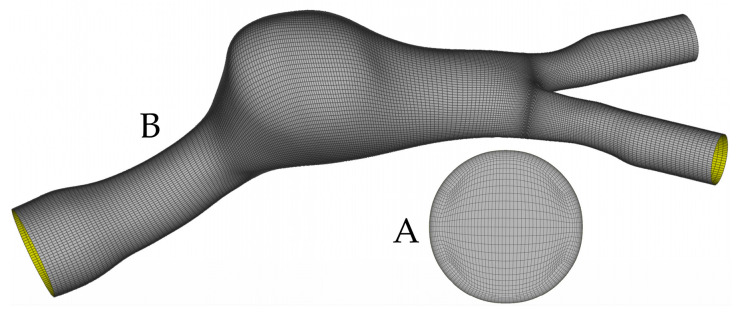
(**A**): Inlet mesh and O-Grid for the construction of boundary layer. (**B**): Surface mesh of the aneurysm and bifurcation area, where it can be seen that the mesh density increases while moving close to the bifurcation, in order to capture non-trivial flow dynamics.

All surfaces were then meshed with ANSA (BETA CAE Systems S.A.) using a pure hexahedral mesh. As shown by De Santis et al. [29], hexahedral meshes should be preferred to other types of meshes since they require a fewer number of elements for a specific level of accuracy. An appropriate O-Grid (with 0.25 parametric and 0.95 Bell shape values) was constructed in all cases to capture the high velocity gradients due to the boundary layer close to the rigid wall. Figure 1 presents the inlet (A) and part of the surface (B) meshes.

### 2.2. Simulation Setup, Boundary Conditions, and Rheology Models

Blood was modeled as an incompressible, homogeneous, and non-Newtonian fluid. The fluid domain was governed by the coupled system of Navier–Stokes and continuity equations formulated as

(1) $$\rho \left[\frac{\partial U}{\partial t}+\left(U\cdot \nabla \right)U\right]=-\nabla P+\nabla \cdot \tau ,\;\nabla \cdot U=0,$$

where the velocity and pressure fields **U**,P, respectively, depended on blood density ρ and stress tensor τ. For the needs of this study, the stress tensor was expressed in terms of the rate-of-deformation tensor **D** and the shear rate $\dot{\gamma }$ as follows

(2) $$\tau =2\;\mu \left(\dot{\gamma }\right)\;D.$$

**Figure 2:**
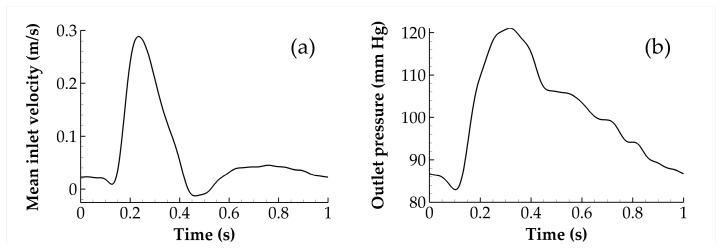
Mean inlet velocity (**a**) and outlet pressure (**b**) for all simulations considered in this study.

All vascular flows were simulated using a commercial finite volume solver (Fluent 17.2, ANSYS Inc., Canonsburg, PA, USA) with a default criterion for convergence equal to 10^−4^. The SIMPLEC algorithm was chosen for pressure–velocity coupling and a fixed time step of 0.005 s was adopted for a cardiac cycle of 1 s. In order to ensure that all transient effects were washed-out, four cycles were simulated before results were collected. All simulations assumed a rigid wall and the no-slip condition was prescribed at the wall boundary. Furthermore, a transient inlet velocity and outlet pressure were prescribed in all simulations; both profiles (Figure 2) closely follow Olufsen et al. [30].

**Figure 3:**
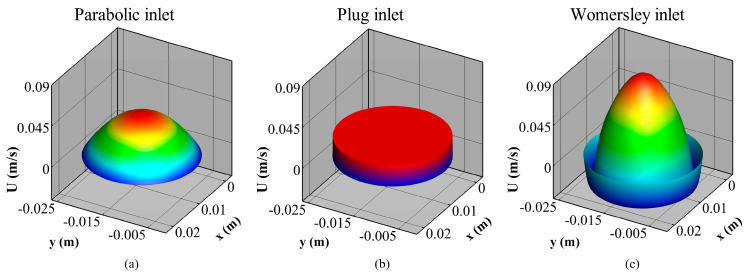
Parabolic (**a**), Plug (**b**), and Womersley (**c**) inlet velocity distribution profiles at the beginning of the cardiac cycle.

The pressure distribution was kept constant for each time step at the outlets. Throughout the cardiac cycle, though, the distribution followed the one shown in Figure 2b. Regarding velocity, three different profiles (Figure 3), namely Parabolic (Par), Plug (Plug), and Womersley (Wom) were prescribed at the inlet, with equal mean values for all three of them (Figure 2a). This allows investigation of the effect of inlet velocity distribution at the hemodynamic behavior of patient-specific aneurysm geometries, which constitutes the first goal of this work.

A user-defined function (UDF) was implemented into the solver to assign the proper velocity according to the following expressions.

(3) UPar(r,t)=2 Umean(t)[1−(rR)2], UPlug(r,t)=U(t), UWom(r,t,n)=Re[iρωn ∂P∂z(1−J0(α i3/2 rRn)J0(α i3/2n))einωt].

Equation (3) defines the streamwise velocity distributions at the inlet in terms of the radius, R, angular frequency, ω, pressure gradient along the flow, ∂P/∂z, number of Fourier modes, n, Womersley parameter, $\alpha =R\sqrt{\omega \rho /\mu },$ and Bessel function, $J_{0}.$ For the needs of this study, fourteen modes were used in the Fourier series expansion to ensure that deviations of the constructed profiles did not deviate more than 0.5% with respect to the ones presented in [30]. In all cases, the flow is assumed laminar with a mean and peak Reynolds number as well as Womersley parameter, α, presented in Table 1.

This assumption is in accordance with [31,32,33,34,35], where it was assumed that blood flow is laminar, even during exercise, for asymmetric AAAs [36]. Care must be taken, though, since turbulence might emerge under certain circumstances. As pointed out in [32], severe angulation of the proximal neck can cause strong turbulence, altering the distribution of WSS on the artery wall. Additionally, Khanafer et al. [37] showed that the increased shear stress due to local turbulence can generate further dilation of the aneurysm sac, creating a mechanism for aneurysmal growth and potential rupture.

The second research question that is investigated herein evaluates the effect of the adopted rheological model on vascular flows. Five specifications were implemented (Table 2); it should be stressed that for shear rate values greater than 100 s^−1^, all models converge to the Newtonian case. Specifications were selected following a recent classification of 16 rheology models [38], which revealed a partition in three main homogeneous groups (clusters) and six in total, with the Newtonian model appearing as an outlier. The Carreau–Yasuda and the Casson models were members of the largest cluster, whereas the Power law was the best representative of the second largest cluster and Herschel–Bulkley a satisfactory representative of the third cluster.

### 2.3. Hemodynamic and Flow Parameters

Various parameters have been used in the literature to study vascular flows in terms of the wall shear stress vector (**WWS**) over the cardiac cycle, T. These include the time average wall shear stress (TAWSS, Pa), oscillatory shear index (OSI), and relative residence time (RRT, Pa^−1^), defined by the following expressions

(4) $$\mathrm{TAWSS}=\frac{1}{T}\int_{0}^{T}\left|\mathrm{WSS}\right|\mathrm{dt},\;\mathrm{OSI}=\frac{1}{2}\left(1-\frac{\left|\int_{0}^{T}\mathrm{WSS}\;\mathrm{dt}\right|}{\int_{0}^{T}\left|\mathrm{WSS}\right|\;\mathrm{dt}}\right),\;\mathrm{RRT}∼\frac{1}{\left(1-2\cdot \mathrm{OSI}\right)\cdot \mathrm{TAWSS}}.$$

According to the above definitions, TAWSS quantifies the average tangential force on the wall over the entire cycle but does not provide any insight on the oscillatory nature of the flow. To this end, He et al. [48] introduced the non-dimensional parameter OSI in order to capture the cyclic variation in **WSS**. As a result, uniaxial flows yield OSI=0 while flows with no preferred direction correspond to OSI = 0.5. Finally, Himburg et al. [49] presented RRT to quantify the time that blood resides close to the wall while accounting for the effect of both TAWSS and OSI. The abovementioned variables provide valuable information for various diseased states, such as thrombogenic stimulating environments for TAWSS < 0.4 Pa [50], OSI > 0.3 [49], and RRT > 10 Pa^−1^ [51].

**Figure 4:**
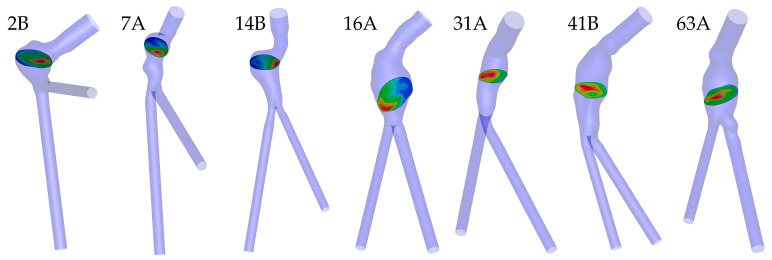
Patient-specific geometries and corresponding planes where flow asymmetry and dispersion were calculated.

An additional set of parameters that characterize flows are flow asymmetry, f_A_, and flow dispersion, f_D_; both quantities were calculated on random planes (to ensure that results were not affected in a systematic way) of the aneurysm sac and quantify the eccentricity and broadness of flows, respectively. Following [18,52], the centroid coordinates $\left(x_{0},\;y_{0},\;z_{0}\right)$ of the top 15% peak systolic velocity $\left(V_{\max}^{15\%}\right)$ were compared with respect to the centroid $\left(x_{c},\;y_{c},\;z_{c}\right)$ of the plane under consideration. Flow asymmetry is formulated as

(5) $$f_{A}\left(\%\right)=100\cdot \frac{\sqrt{{\left(x_{0}-x_{c}\right)}^{2}+{\left(y_{0}-y_{c}\right)}^{2}+{\left(z_{0}-z_{c}\right)}^{2}}}{R_{\mathrm{eq}}},$$

where $R_{\mathrm{eq}}$ is the equivalent radius of a circle with the same area as the plane vessel. Thus, flows with low asymmetry values do not deviate significantly from the plane centroid, while flows with high values are eccentric and develop mainly close to the vessel wall. In a similar manner,

(6) $$f_{D}\left(\%\right)=100\cdot \frac{\mathrm{area}\;\mathrm{of}\;V_{\max}^{15\%}}{\mathrm{area}\;\mathrm{of}\;\mathrm{plane}},$$

yielding a broad velocity profile for large dispersion values and a sharper one as values decrease. Figure 4 depicts the seven patient-specific geometries and the corresponding planes where flow asymmetry and dispersion were calculated.

### 2.4. Statistical Analysis

The effects of alternative inlet velocity distributions (Figure 3) and rheological models (Table 2) at the hemodynamic behavior of real aneurysm geometries was investigated with two-way ANOVA tests, which evaluate simultaneously the effect of two grouping variables (or factors) on a response variable [53]. The level combinations of the two grouping variables, denoted by IVD and R_m_, respectively, produced a balanced design, as the sample sizes within each level of the independent factors are equal. Fourteen response variables that characterize vascular flows were examined: flow asymmetry (FA_pct_), flow dispersion (FD_pct_), percentage areas with thrombus-prone conditions, designated by the levels of TAWSS (TAWSS_pct_: % area with TAWSS < 0.4 Pa), OSI (OSI_pct_: % area with OSI > 0.3), and RRT (RRT_pct_: % area with RRT > 10 Pa^−1^), and the observed average levels, minima and maxima of hemodynamic variables, namely TAWSS (TAWSS_ave_, TAWSS_min_, TAWSS_max_), OSI (OSI_ave_, OSI_min_, OSI_max_), and RRT (RRT_ave_, RRT_min_, RRT_max_).

Two-way ANOVA designs evaluate the following initial (null) hypotheses per response variable [53]: (a) there are no significant differences in the observed means across the three alternative IVD choices; (b) there are no significant differences in the observed means across the five alternative rheological models; (c) there is no significant interaction between IVD and R_m_. If an ANOVA test signifies substantial evidence against the null hypothesis of equal means across factor levels, the analysis proceeds in the second stage, which comprises multiple pairwise comparisons between the group means: the latter determine if specific group pairs (for R_m_, say P versus N, or for IVD, Plug versus Parabolic) are significantly different. On the other hand, if the *p*-values from an ANOVA test do not provide evidence against the null hypothesis of equal means across groups, there is no need to conduct a post hoc test to determine which groups are different from each other.

Conventional ANOVA is based on group means and assumes normality and homoscedasticity (equal variances across groups) for model residuals. Violation of the abovementioned assumptions may yield inaccurate confidence intervals and poor characterization regarding the significance of observed group differences [54]. Preliminary analyses of the data presented in the next section revealed strong evidence against the assumption of homoscedastic residuals. To safeguard quantitative analyses from false hypotheses, we adopted trimmed-mean-based methods, which are robust to deviations from normality and homoscedasticity. Specifically, the robust two-way ANOVA procedure and the corresponding post hoc tests presented in (p. 335, [54]) were implemented, using R package WRS2 [55]. In the post hoc comparisons, both confidence intervals and *p*-values were adjusted to account for multiple testing by controlling the family-wise error rate (or the probability of false significance), using the Hochberg method [54].

The null hypotheses in the statistical tests imply that the main characteristics of vascular flows do not differ substantially for alternative IVD and R_m_ choices. In what follows, to evaluate evidence that favors the alternative hypothesis, we use *p*-values following the recent recommendation in [56]: values between 0.005 and 0.05 offer weak or “suggestive” evidence, whereas values lower than 0.005 provide strong evidence against the null hypothesis. It should be stressed that *p*-values are often misinterpreted in ways that lead to overstating the evidence against the null hypothesis when conventional thresholds (e.g., *p*-value = 0.05) that signify “statistical significance” are utilized [57]. As shown in [56], conventional levels of significance do not actually provide strong evidence against the null hypothesis. When the prior probabilities of the null and the alternative hypotheses are equal, the upper bound on the posterior probability of the alternative hypothesis equals 0.89 for a *p*-value of 0.01, which is often considered “highly significant”; hence, there remains at least an 11% chance that the null hypothesis is true. For a *p*-value of 0.005, the upper bound on the posterior probability of the alternative hypothesis equals 0.933, which reduces the chance that the null is true by about 50% relative to when the *p*-value equals 0.01 [56].

## 3. Results

### 3.1. Mesh Convergence

The well-established Grid Convergence Index (GCI) method [58] was adopted in order to access the discretization error of all simulations presented herein. To this end, three simulations with constant refinement ratio r=2 were performed for the 31A case of the Casson model with Parabolic profile. Denoting $f_{1},\;f_{2},\;f_{3}$ the solutions for the fine, medium, and coarse mesh respectively of a parameter of interest, the order of convergence p equals [58]

(7) $$p=\ln\left(\frac{f_{3}-f_{2}}{f_{2}-f_{1}}\right)/\ln\left(r\right).$$

The GCI between two successive mesh refinements i and j can then be formulated as

(8) $$\mathrm{CG}I_{\mathrm{ij}}=\frac{F_{s}\cdot e_{\mathrm{ij}}}{r^{p}-1},$$

where the safety factor $F_{s}$ is usually set equal to 1.25 [59], and $e_{\mathrm{ij}}$ is the relative error of f. Finally, convergence is achieved when the ratio $\mathrm{CG}I_{23}/\left(r^{p}\cdot \mathrm{CG}I_{12}\right)$ satisfies the following condition

(9) $$\frac{\mathrm{CG}I_{23}}{r^{p}\cdot \mathrm{CG}I_{12}}\to 1.$$

Convergence was accessed by examining the three hemodynamic variables of interest, namely TAWSS, OSI, and RRT. The final mesh consisted of 776,000 first-order hexahedral elements (reference mesh). All simulations were performed using meshes with a total number of elements that do not vary more than 5% with respect to the reference mesh. Table 3 presents the corresponding results; one can observe convergence rates well within the acceptable regime for grid convergence.

### 3.2. ANOVA Models

**Figure 5:**
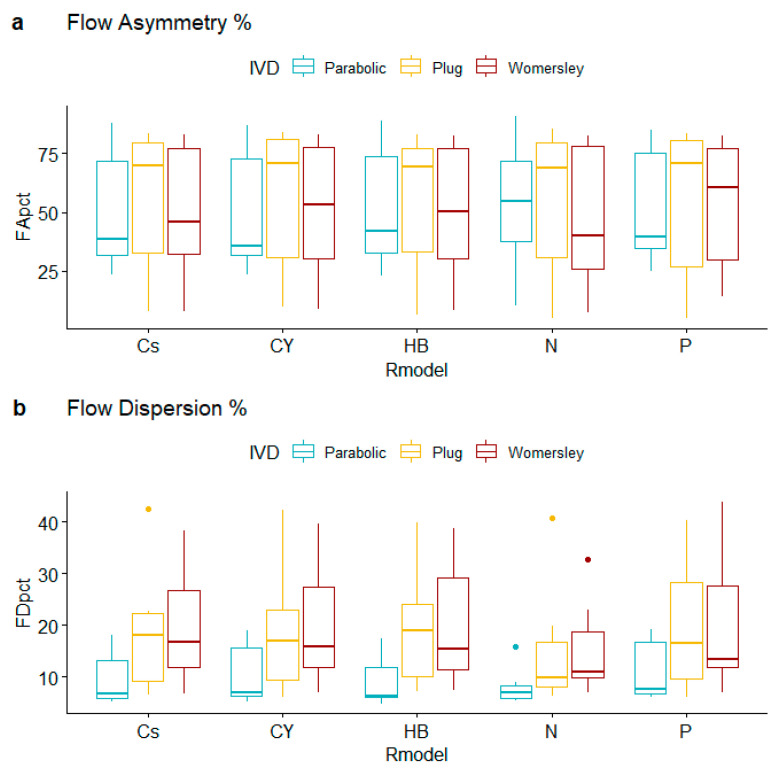
Flow asymmetry and dispersion boxplots for alternative rheological models and inlet velocity distributions.

Table 4 summarizes the findings of 14 robust, trimmed-mean-based ANOVA models, which evaluate the effects of alternative inlet velocity distributions and rheological models regarding the hemodynamic behavior of real aneurysm geometries. It is worth highlighting that the observed *p*-values clearly suggest that interaction effects are negligible; hence, the effects of IVD do not depend on the selected rheological specification and vice versa, for all response variables examined. Starting with flow asymmetry (Table 5), there is no evidence of different average levels across IVD or R_m_ groups. Figure 5a supports this finding, with substantial overlapping in the group-specific boxplots.

On the other hand, observed flow dispersion percentages differ across IVD specifications (Figure 5b), with the corresponding post hoc comparisons suggesting that it is the Parabolic inlet velocity specification that produces lower average levels for FD_pct_. Specifically, the difference in FD_pct_ trimmed-means equals −6.982 for Parabolic vs. Plug (95% CI [−12.674, −1.289]); the corresponding *p*-value equals 0.012, which provides weak evidence against the null hypothesis of equal average levels. A noteworthy, although also weak, dissimilarity is also observed between Parabolic and Womersley specifications: the difference in FD_pct_ trimmed-means equals −8.314 (95% CI [−15.626, −1.003]) and the corresponding *p*-value = 0.015.

**Figure 6:**
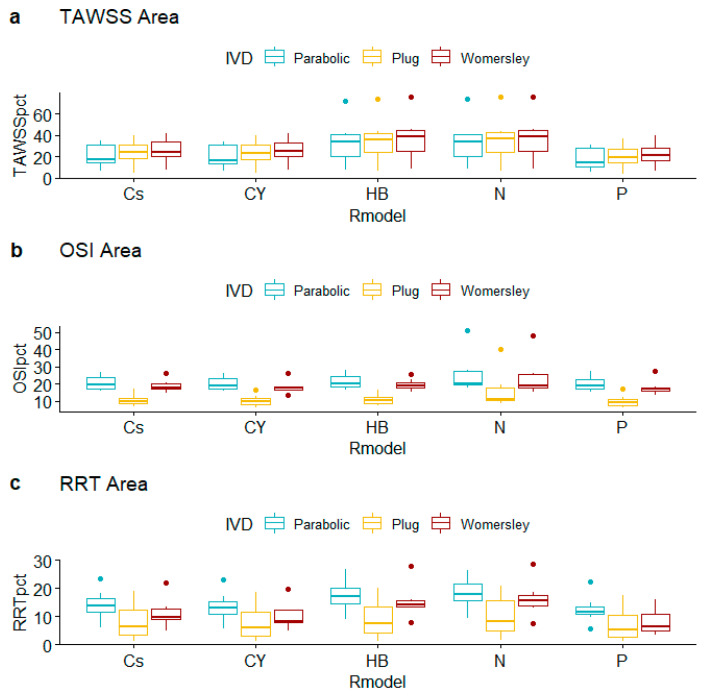
Boxplots of thrombogenic region percentages for alternative rheological models and inlet velocity distributions.

Regarding thrombus-prone percentage areas (Table 6), it is interesting to observe that TAWSS-based assessments depend weakly on the selected rheological model, whereas OSI-based assessments depend strongly on the chosen inlet velocity distribution (Figure 6). The post hoc comparisons suggest that the observed differences for TAWSSpct are mainly due to N vs. P and HB vs. P (N vs. P difference in TAWSSpct equals 14.069, CI [0.299, 27.88839], *p*-value = 0.044; HB vs. P difference in TAWSSpct equals 13.956, CI [0.191, 27.722], *p*-value = 0.044; both *p*-values provide weak evidence against the null hypothesis of equal means across groups).

Observed differences in OSI-defined thrombus-prone percentage areas provide very strong evidence against the null hypothesis for the specification pairs Plug vs. Womersley (difference in OSI_pct_ equals −7.920, CI [−10.313, −10.313], *p*-value < 0.001) and Plug vs. Parabolic (difference in OSI_pct_ equals 10.109, CI [6.914, 13.304], *p*-value < 0.001). This is clearly observed in Figure 6b: the distributions of OSI_pct_ computed when the Plug specification is adopted lie clearly below the ones from Womersley and Parabolic. On the other hand, there is no evidence of different outcomes for Parabolic vs. Womersley distribution profiles (difference in OSI_pct_ equals 2.189, CI [−1.017, 5.396], *p*-value = 0.096). RRT-based assessments differ mainly in the IVD factor pair Parabolic vs. Plug (difference in RRT_pct_ equals 6.997, CI [3.092, 10.902], *p*-value < 0.001) with weak evidence against the null hypothesis for N vs. P rheologies (difference in RRT_pct_ equals 6.083, CI [0.133, 12.032], *p*-value = 0.046).

**Figure 7:**
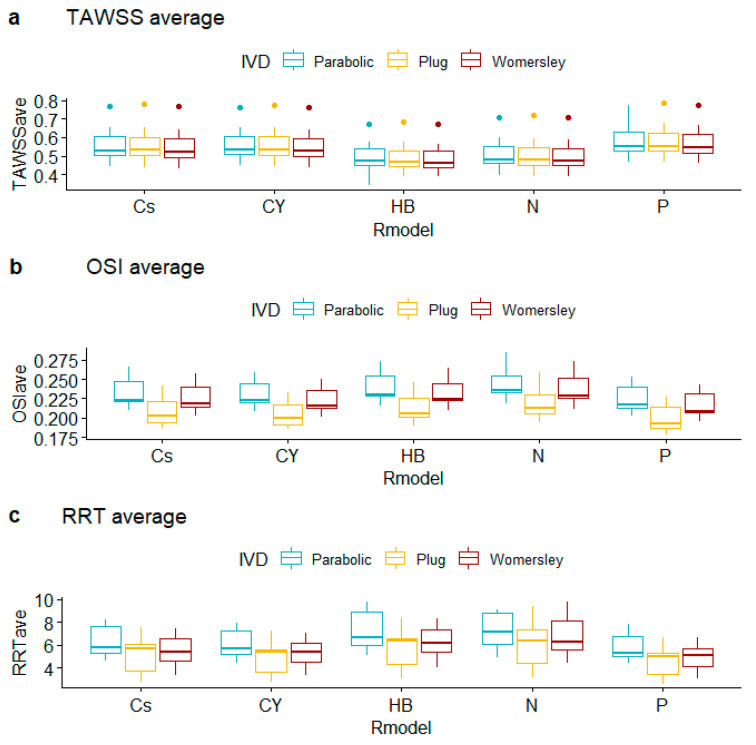
Boxplots of averages of hemodynamic variables for alternative rheological models and inlet velocity distributions.

**Figure 8:**
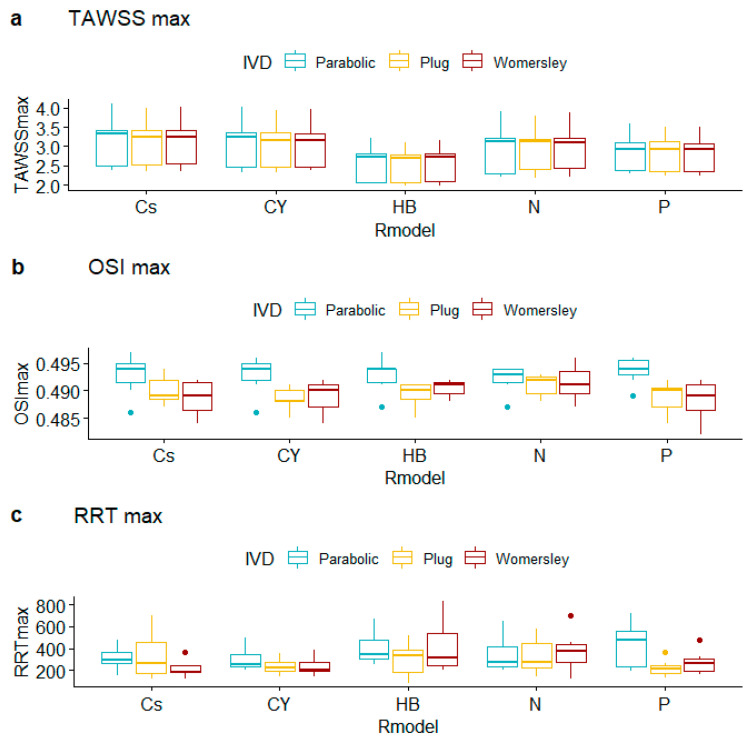
Boxplots of observed maxima of hemodynamic variables for alternative rheological models and inlet velocity distributions.

**Figure 9:**
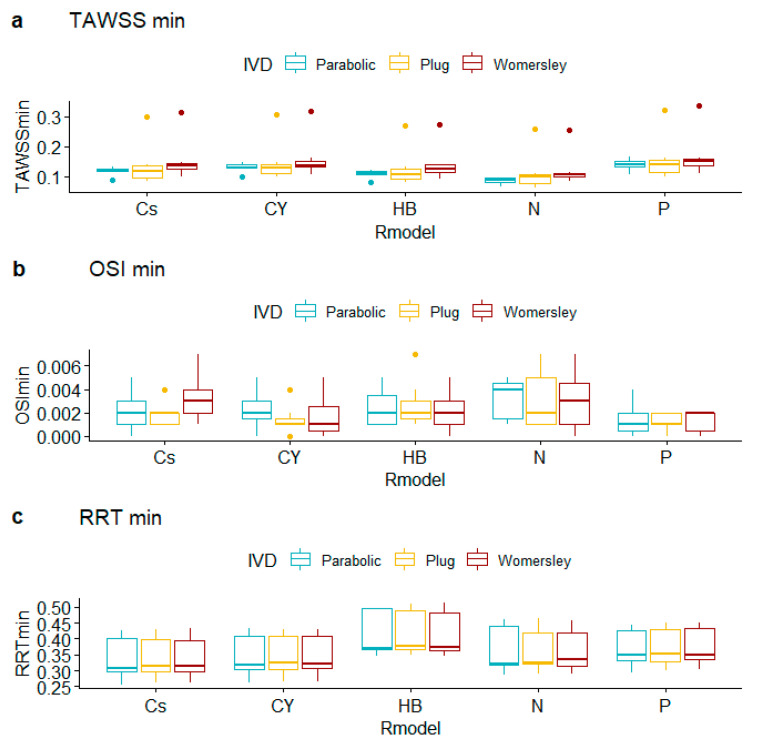
Boxplots of observed minima of hemodynamic variables for alternative rheological models and inlet velocity distributions.

TAWSS summary metrics (Table 7, Table 8 and Table 9, Figure 7, Figure 8 and Figure 9) do not differ substantially for alternative rheological specifications; calculated TAWSSmin is an exception due to the following pairs: N vs. P (difference in TAWSSmin equals −0.047, CI [−0.070, −0.023], *p*-value < 0.001), CY vs. N (difference in TAWSSmin equals 0.037, CI [0.020, 0.055], *p*-value < 0.001), and Cs vs. N (difference in TAWSSmin equals 0.028, CI [0.009, 0.047], *p*-value = 0.001); all *p*-values provide strong evidence against the null hypothesis. Interestingly, the observed differences in average levels of TAWSSave and TAWSSmax were essentially statistically indistinguishable.

Observed OSI averages depend on IVD with strong evidence for different outcomes for the pairs Plug vs. Parabolic (difference in OSI_ave_ equals 0.026, CI [0.012, 0.041], *p*-value < 0.001) and Plug vs. Womersley (difference in OSI_ave_ equals −0.019, CI [−0.032, −0.005], *p*-value = 0.003). Observed OSI maxima differ for the pairs Parabolic vs. Plug (difference in OSI_max_ equals 0.004, CI [0.002, 0.005], *p*-value < 0.001) and Parabolic vs. Womersley (difference in OSI_max_ equals 0.004, CI [0.002, 0.005], *p*-value < 0.001). Interestingly, the observed differences in average levels of OSI_min_, RRT_ave_, RRT_min_, and RRT_max_ do not provide evidence against the null hypothesis, for all R_m_ and IVD specifications examined. The pairwise tests provided very weak evidence against the null hypothesis of equal RRT_ave_ means across groups for the Parabolic vs. Plug IVD pair (difference in RTT_ave_ equals 1.241, CI [0.005, 2.478], *p*-value = 0.05).

## 4. Discussion

The above results carry several implications for methodologies developed to answer clinical questions. Computational modeling should provide insights regarding the physiology of the circulatory system, pathophysiology of cardiovascular diseases, and performance of vascular therapies. If one aims to predict thrombus deposition in order to characterize the risk profile of a given AAA, such a prediction needs to be accurate in order to be relevant from a clinical perspective. In other words, if therapeutic decisions and management of these lesions are going to be determined based on computational modeling and numerical simulations, these must provide sound rupture risk estimation.

Hemodynamic variables may exert direct clinical implications during AAAs evaluation, related to thrombus-prone regions and relevant tangential forces. Indeed, since intraluminal thrombus has been previously shown to be a significant determinant of the risk of rupture, the choice of the inlet profile or rheological model may affect AAA assessment. An investigation of the literature suggests that different rheological models and inlet velocity distribution profiles are used interchangeably in computational modeling, with uncertain implications for the results obtained. Deviations between different model assumptions should be recorded, and reporting standards regarding the methodology of such calculations should be developed.

The null hypotheses examined here essentially imply that practitioners with different choices regarding their simulation setups ought to produce vascular flows that do not differ substantially in terms of their main characteristics: TAWSS, OSI, percentages of thrombus-prone regions, flow asymmetry, and dispersion. The outlier- and heteroscedasticity-robust statistical tests presented in Section 3 (which are conservative, relative to conventional, non-robust procedures) suggest that RRT, a variable of primary interest as it takes into account both the average tangential force on the wall over the entire cycle and the oscillatory nature of the flow, does not differ significantly across rheological models and inlet velocity distribution profiles. The same finding is also observed when one focuses on flow asymmetry, whereas differences in TAWSS levels are mainly due to the Newtonian specification; non-Newtonian alternatives that are widely applied in practice do not lead to strong evidence against the null hypothesis of equal levels.

Interestingly, our analyses suggest that the selection of an inlet velocity profile appears as a more critical factor relative to the rheological specification in vascular flow simulations. Furthermore, the two factors exert additive effects on variables that characterize vascular flows, with negligible interaction effects. Indeed, flow dispersion averages derived using the Parabolic profile are substantially lower relative to the ones corresponding to Plug or Womersley specifications. On the other hand, observed differences are statistically indistinguishable for alternative rheological models. If one focuses on thrombus-prone regions and the oscillatory characteristics of the flow (via OSI), it is mainly the Plug specification that produces substantially lower percentages relative to Parabolic and Womersley profiles.

Taking into account previous studies, one could assume that a Parabolic or Womersley inlet profile approximates patient-specific flow conditions more closely compared to the Plug profile, at least for critical hemodynamic variables such as thrombus-prone AAA segments [18,24]. Conversely, focusing on other variables such as flow dispersion, other patterns can be identified, such as the Parabolic profile being the specification that produced heterogeneity. Nevertheless, the relevance of this metric and the clinical implications of large versus small flow dispersion are not yet well understood. Patient-specific inflow velocity profiles might be advantageous, but these would require advanced imaging modalities and analysis, which can be challenging, impractical, and not always available.

As with the majority of studies, the present work is subject to limitations. The first one is the rigid wall assumption, neglecting the effect of wall compliance. A second limitation is the implementation of only one outlet pressure boundary condition. It should be noted though that due to the large number of rheology models and patient-specific geometries, it was practically not possible to incorporate the above two additional factors since the required simulations would have been in the order of thousands. To overcome this obstacle, a future work will include fewer geometries and blood flow models, which according to the results of this study are less critical with respect to the choice of inlet velocity profile.

## 5. Conclusions

This manuscript evaluates the effects of alternative modeling choices, namely alter-native rheological specifications and inlet velocity distributions, on simulated blood flows. The finite volume method was employed, focusing on seven patient-specific abdominal aortic aneurysms. Fourteen variables that characterize and summarize vascular flows were computed; for each case, five rheological specifications and three inlet velocity boundary conditions were utilized. The significance of observed differences, which result from different modeling choices, were evaluated to our knowledge for the first time, with modern and robust statistical procedures.

Our findings can be summarized as follows. The two examined factors, namely rheological models and inlet velocity distributions, exert additive effects; to put it otherwise, their interaction effects were negligible on all variables that characterize vascular flows. The selected inlet velocity profile is a stronger factor, relative to the chosen rheology, for variables that are associated with the oscillatory characteristics of blood flow. Interestingly, response variables that relate to the average tangential force on the wall over the entire cycle do not differ significantly across alternative factor levels, as long as one focuses on non-Newtonian specifications.

## Figures and Tables

**Table 1:** Mean and peak inlet, Re, as well as Womersley parameter, α.

Case	Inlet Radius (m)	Mean Re	Max Re	α
2B	0.01148	452.0	2026.5	15.87
7A	0.01167	459.4	2060.0	16.13
14B	0.01168	459.8	2061.8	16.15
16A	0.01157	455.5	2042.4	16.00
31A	0.01157	455.5	2042.4	16.00
41B	0.01152	453.5	2033.5	15.93
63A	0.01145	450.8	2021.2	15.83

**Table 2:** Rheology models and parameter values.

Name (Abbreviation)	Expression	Parameter Values	References
Carreau–Yasuda (CY)	$\mu \left(\dot{\gamma }\right)=\mu_{\infty }+\frac{\left(\mu_{0}-\mu_{\infty }\right)}{\left[1+{\left(\lambda \dot{\gamma }\right)}^{\alpha }\right]{\;}^{\left(1\;-\;n\right)/\alpha }}$	$\begin{array}{l} \;\;\mu_{\infty }=0.00345,\;\mu_{0}=0.056\\ \lambda =1.902,\;n=0.22,\;\alpha =1.25 \end{array}$	[14,39,40]
Casson (Cs)	$\mu \left(\dot{\gamma }\right)={\left(\sqrt{\;k}+\frac{\sqrt{\;\tau_{0}}}{\sqrt{\;\dot{\gamma }}}\right)}^{2}$	$k=0.00345,\;\tau_{0}=0.005$	[39,41]
Herschel–Bulkley (HB)	$\mu \left(\dot{\gamma }\right)=k\;{\dot{\gamma }}^{n-1}+\frac{\tau_{0}}{\dot{\gamma }}$	$\begin{array}{l} \tau_{0}=0.00345,\;k=0.008\\ \;\;\;\;n=0.8375 \end{array}$	[10,42]
Newtonian (N)	$\mu \left(\dot{\gamma }\right)=\mu_{\infty }$	$\mu_{\infty }=0.00345$	[43,44]
Power law (P)	$\mu \left(\dot{\gamma }\right)=k\;{\dot{\gamma }}^{n-1}$	k=0.01467, n=0.7755	[45,46,47]

**Table 3:** Convergence Grid Index for the 31A, Cs, Parabolic case, and convergence rates for TAWSS, OSI, and RRT.

# Elements	194,392	388,080	776,000	p	$CGI_{12}$	$CGI_{23}$	$CGI_{23}/\left(r^{p}CGI_{12}\right)$
TAWSS	0.6507	0.6523	0.6530	1.25533	0.093225	0.222779	1.0010
OSI	0.2209	0.2227	0.2231	2.11008	0.069600	0.301029	1.0019
RRT	5.0077	5.1355	5.1875	1.29873	0.857794	2.131632	1.0101

**Table 4:** *p*-values derived from heteroscedasticity- and outlier-robust, trimmed-mean-based ANOVA analyses for the effects of alternative inlet velocity distributions and rheological models at the hemodynamic behavior of real aneurysm geometries. Response variables related to hemodynamic behavior are shown in rows with the two main factors and their interaction in columns. *p*-values very close to zero, shown in bold, provide strong evidence against the null hypothesis of equal means across factor groups.

	R_m_	IVD	R_m_:IVD
FA%	0.999	0.719	0.999
FD%	0.415	0.002	0.999
TAWSS%	0.009	0.392	0.999
OSI%	0.237	0.001	0.999
RRT%	0.008	0.001	0.987
TAWSSave	0.030	0.916	0.999
OSIave	0.065	0.001	0.999
RRTave	0.045	0.017	0.999
TAWSSmax	0.072	0.999	0.999
OSImax	0.671	0.001	0.691
RRTmax	0.166	0.201	0.659
TAWSSmin	0.001	0.016	0.996
OSImin	0.104	0.710	0.890
RRTmin	0.108	0.995	0.999

**Table 5:** Percentage values for flow asymmetry and dispersion.

Peak Systole		Flow Asymmetry					Flow Dispersion				
		Cs	CY	HB	N	P	Cs	CY	HB	N	P
2B	Parabolic	38.45	32.87	42.09	39.87	32.08	5.11	5.22	4.79	5.94	6.28
	Plug	79.61	83.54	74.70	78.37	83.02	7.05	5.99	8.90	7.75	6.03
	Womersley	46.05	53.12	50.48	30.63	60.54	16.78	15.85	15.43	10.29	13.34
7A	Parabolic	57.87	60.21	61.78	57.14	65.57	5.87	6.31	6.06	6.97	6.94
	Plug	69.90	71.08	69.35	68.82	71.07	17.96	16.94	19.00	19.73	16.48
	Womersley	73.84	73.97	74.44	74.70	73.72	12.91	13.79	12.95	10.87	12.79
14B	Parabolic	85.62	85.24	85.76	86.72	84.88	5.79	6.03	5.87	5.42	6.10
	Plug	83.75	83.85	83.28	85.32	83.36	6.53	6.60	7.19	6.21	6.89
	Womersley	82.90	83.14	82.60	82.39	82.65	6.75	6.83	7.31	6.97	6.99
16A	Parabolic	88.11	87.08	88.77	90.74	84.92	6.63	6.97	6.29	5.64	7.61
	Plug	79.80	79.12	80.12	80.64	78.19	11.24	12.07	11.24	8.50	12.20
	Womersley	80.87	81.48	80.37	81.59	80.46	10.82	9.90	9.84	9.53	10.89
31A	Parabolic	34.44	35.62	34.59	35.67	37.35	18.06	19.03	17.31	15.91	19.13
	Plug	35.06	36.10	36.25	34.27	36.81	42.48	42.30	39.95	40.81	40.41
	Womersley	35.38	37.43	40.41	39.89	37.44	38.37	39.65	35.53	32.68	36.35
41B	Parabolic	28.96	30.63	30.40	54.89	39.39	17.29	17.83	14.86	7.44	17.85
	Plug	30.10	24.99	29.96	27.29	16.99	22.64	23.49	26.74	9.87	36.70
	Womersley	29.15	22.94	19.83	20.77	22.09	32.74	35.03	38.73	14.63	43.93
63A	Parabolic	23.69	23.50	22.99	10.07	25.16	9.07	13.15	8.56	8.96	15.48
	Plug	7.69	9.67	6.59	4.91	4.87	21.77	22.26	21.56	13.53	19.72
	Womersley	7.61	8.75	8.15	7.26	13.94	20.78	19.83	22.89	22.97	18.90

**Table 6:** Percentage areas with thrombus-prone conditions.

		% Area with TAWSS < 0.4 Pa					% Area with OSI > 0.3					% Area with RRT > 10 Pa^−1^				
		Cs	CY	HB	N	P	Cs	CY	HB	N	P	Cs	CY	HB	N	P
2B	Parabolic	16.74	15.78	24.27	25.29	12.06	17.10	16.99	17.20	19.82	17.34	12.34	11.76	15.21	16.78	11.74
	Plug	23.97	23.52	27.47	27.98	19.00	6.686	6.257	7.248	9.636	6.135	3.778	3.438	4.792	4.810	2.801
	Womersley	24.34	24.75	27.86	27.58	20.75	18.78	18.52	18.92	18.59	18.12	9.646	7.513	15.09	16.20	4.266
7A	Parabolic	17.14	16.50	72.52	74.26	14.04	20.25	19.98	21.52	51.21	19.72	13.68	13.07	17.54	17.85	12.42
	Plug	20.73	20.23	74.24	75.87	17.03	10.08	9.522	10.99	40.00	9.337	6.328	5.990	7.590	8.158	5.203
	Womersley	21.93	21.87	75.82	75.99	19.21	17.88	17.47	18.80	47.79	17.56	9.891	8.384	13.70	14.16	5.767
14B	Parabolic	6.703	6.420	7.536	8.149	5.482	15.96	15.74	16.74	17.56	15.35	6.121	5.616	8.872	9.348	5.575
	Plug	4.920	4.483	6.405	6.670	3.220	9.223	9.037	9.428	10.99	8.367	1.400	1.392	1.382	1.759	1.369
	Womersley	7.594	7.446	8.310	8.411	6.815	16.02	15.94	16.75	17.31	15.07	5.050	4.969	8.009	7.514	3.603
16A	Parabolic	30.67	30.54	33.91	34.32	27.94	17.42	16.75	18.81	20.31	16.58	14.14	13.45	16.99	18.42	11.50
	Plug	31.82	31.32	36.25	36.74	28.69	10.02	9.818	10.15	10.55	9.889	12.61	12.10	13.86	15.73	10.62
	Womersley	34.05	33.44	38.53	38.78	30.06	14.37	13.70	15.23	15.29	13.30	11.97	12.00	14.16	15.61	10.58
31A	Parabolic	11.66	11.44	16.58	15.71	9.094	19.45	19.16	19.80	19.72	19.11	10.83	10.14	14.20	14.27	9.897
	Plug	14.89	13.71	21.33	21.05	10.63	7.300	6.747	7.570	8.490	6.607	3.100	2.941	3.857	4.802	2.950
	Womersley	18.14	17.83	22.86	22.84	13.99	17.58	17.26	18.38	18.57	17.03	8.299	7.894	13.28	13.21	6.488
41B	Parabolic	31.07	30.68	41.50	41.10	27.22	26.66	26.00	27.96	28.10	25.21	18.42	17.17	22.60	24.36	14.79
	Plug	30.75	29.74	40.77	41.60	26.20	13.63	12.84	13.77	15.79	12.40	11.74	10.75	13.07	15.90	10.09
	Womersley	33.64	31.79	45.37	45.39	26.13	20.90	18.34	22.49	25.27	16.78	13.57	12.37	15.90	18.55	10.97
63A	Parabolic	34.81	34.00	41.01	40.22	31.08	26.73	26.29	27.41	26.26	27.45	23.36	22.88	26.78	26.35	22.33
	Plug	40.31	39.84	43.62	43.44	36.81	16.85	16.66	16.64	19.41	17.07	19.10	18.46	20.09	20.77	17.37
	Womersley	42.09	41.95	44.94	44.85	40.02	26.04	26.40	25.68	26.25	27.32	22.12	19.81	27.81	28.78	15.87

**Table 7:** Average values and TAWSS, OSI, and RRT.

Average Values		TAWSS					OSI					RRT				
		Cs	CY	HB	N	P	Cs	CY	HB	N	P	Cs	CY	HB	N	P
2B	Parabolic	0.558	0.561	0.498	0.502	0.586	0.221	0.219	0.228	0.236	0.213	5.363	5.204	6.236	6.562	5.012
	Plug	0.548	0.550	0.486	0.494	0.574	0.196	0.193	0.202	0.208	0.187	4.075	3.893	4.678	4.742	3.629
	Womersley	0.546	0.546	0.487	0.496	0.571	0.218	0.215	0.224	0.229	0.208	4.801	4.673	5.599	5.689	4.241
7A	Parabolic	0.443	0.447	0.340	0.397	0.467	0.266	0.259	0.273	0.285	0.253	7.685	7.341	9.214	9.063	7.119
	Plug	0.438	0.443	0.392	0.392	0.466	0.241	0.233	0.246	0.259	0.227	5.702	5.423	6.419	6.986	5.030
	Womersley	0.433	0.438	0.389	0.390	0.460	0.257	0.250	0.264	0.273	0.243	6.297	5.938	7.199	7.603	5.487
14B	Parabolic	0.765	0.761	0.674	0.708	0.773	0.210	0.208	0.216	0.218	0.203	4.584	4.464	5.071	4.882	4.457
	Plug	0.777	0.773	0.684	0.718	0.784	0.186	0.185	0.190	0.194	0.178	2.783	2.761	3.097	3.144	2.607
	Womersley	0.767	0.763	0.674	0.710	0.775	0.202	0.201	0.209	0.211	0.195	3.388	3.327	4.065	4.417	3.079
16A	Parabolic	0.530	0.532	0.471	0.480	0.553	0.222	0.220	0.229	0.233	0.213	5.781	5.703	6.627	7.135	5.298
	Plug	0.531	0.533	0.470	0.480	0.554	0.202	0.200	0.206	0.212	0.193	5.987	5.408	6.398	6.375	5.054
	Womersley	0.524	0.526	0.464	0.473	0.547	0.213	0.212	0.220	0.223	0.206	5.364	5.390	6.123	6.230	5.065
31A	Parabolic	0.653	0.652	0.575	0.597	0.670	0.223	0.222	0.230	0.232	0.217	5.188	5.106	5.731	5.532	4.977
	Plug	0.654	0.655	0.573	0.593	0.675	0.192	0.190	0.199	0.203	0.185	3.457	3.364	3.897	4.022	3.243
	Womersley	0.644	0.644	0.566	0.585	0.663	0.215	0.214	0.224	0.227	0.207	4.380	4.265	5.237	5.407	3.922
41B	Parabolic	0.510	0.512	0.454	0.463	0.532	0.247	0.244	0.254	0.257	0.239	7.573	7.145	8.525	8.727	6.453
	Plug	0.515	0.518	0.457	0.465	0.540	0.216	0.212	0.220	0.226	0.208	6.203	5.817	6.791	7.595	5.536
	Womersley	0.505	0.508	0.448	0.455	0.529	0.235	0.230	0.241	0.249	0.225	6.829	6.324	7.452	9.815	5.893
63A	Parabolic	0.499	0.501	0.445	0.458	0.519	0.248	0.245	0.254	0.253	0.242	8.257	7.884	9.734	8.800	7.831
	Plug	0.486	0.489	0.431	0.438	0.509	0.226	0.223	0.230	0.235	0.219	7.532	7.224	8.326	9.404	6.685
	Womersley	0.484	0.486	0.431	0.439	0.506	0.244	0.242	0.249	0.253	0.237	7.446	7.035	8.267	8.589	6.653

**Table 8:** Maximum values and TAWSS, OSI, and RRT.

Maximum Values		TAWSS					OSI					RRT				
		Cs	CY	HB	N	P	Cs	CY	HB	N	P	Cs	CY	HB	N	P
2B	Parabolic	2.389	2.337	2.049	2.207	2.299	0.490	0.491	0.491	0.493	0.492	152.1	227.5	348.6	223.3	271.9
	Plug	2.438	2.332	1.971	2.356	2.235	0.487	0.488	0.491	0.493	0.489	260.5	178.0	338.5	247.6	162.1
	Womersley	2.511	2.398	2.080	2.426	2.244	0.485	0.491	0.489	0.492	0.482	185.3	387.2	836.6	290.3	268.0
7A	Parabolic	3.467	3.348	2.790	3.249	3.149	0.495	0.493	0.497	0.494	0.496	249.6	200.0	426.6	202.7	476.4
	Plug	3.440	3.363	2.814	3.193	3.151	0.489	0.488	0.488	0.488	0.490	203.0	203.2	203.5	190.4	262.3
	Womersley	3.393	3.328	2.818	3.095	3.099	0.491	0.487	0.491	0.490	0.489	186.4	180.8	217.6	412.7	166.6
14B	Parabolic	4.098	4.010	3.227	3.888	3.600	0.497	0.495	0.492	0.491	0.495	419.9	326.4	523.0	392.8	596.1
	Plug	3.998	3.945	3.097	3.783	3.498	0.488	0.490	0.485	0.488	0.490	118.7	223.9	85.62	138.5	128.6
	Womersley	4.010	3.952	3.145	3.860	3.496	0.488	0.490	0.491	0.496	0.490	124.8	141.7	389.7	454.1	199.3
16A	Parabolic	3.369	3.352	2.726	3.140	3.045	0.486	0.486	0.487	0.487	0.489	295.7	242.1	258.0	274.6	191.3
	Plug	3.409	3.362	2.766	3.180	3.076	0.494	0.488	0.491	0.492	0.484	701.2	253.6	516.4	579.2	218.0
	Womersley	3.447	3.350	2.813	3.305	3.021	0.484	0.487	0.488	0.487	0.485	231.4	244.3	279.8	120.9	477.8
31A	Parabolic	2.598	2.560	2.029	2.380	2.439	0.494	0.495	0.494	0.494	0.496	301.9	359.8	327.8	238.3	521.6
	Plug	2.627	2.568	2.141	2.439	2.397	0.491	0.490	0.489	0.492	0.491	150.4	146.5	165.6	270.7	181.5
	Womersley	2.585	2.545	2.109	2.425	2.384	0.492	0.492	0.490	0.491	0.492	174.8	200.6	204.0	263.4	177.5
41B	Parabolic	2.420	2.388	2.056	2.207	2.291	0.495	0.496	0.494	0.494	0.494	284.9	257.4	273.5	441.5	200.2
	Plug	2.363	2.379	2.002	2.194	2.302	0.489	0.485	0.490	0.493	0.485	428.3	297.5	373.5	473.0	227.5
	Womersley	2.354	2.376	1.977	2.214	2.293	0.489	0.484	0.492	0.495	0.488	247.3	203.5	319.8	703.1	276.9
63A	Parabolic	3.317	3.237	2.805	3.198	2.938	0.493	0.494	0.494	0.492	0.494	478.0	493.5	671.8	646.5	724.2
	Plug	3.232	3.155	2.698	3.115	2.919	0.493	0.491	0.491	0.491	0.492	481.5	352.8	394.8	420.5	362.5
	Womersley	3.245	3.165	2.724	3.139	2.921	0.492	0.491	0.492	0.489	0.492	370.2	312.8	690.5	379.0	328.5

**Table 9:** Minimum values and TAWSS, OSI, and RRT.

Minimum Values		TAWSS					OSI					RRT				
		Cs	CY	HB	N	P	Cs	CY	HB	N	P	Cs	CY	HB	N	P
2B	Parabolic	0.131	0.147	0.118	0.089	0.165	0.005	0.003	0.005	0.005	0.004	0.425	0.433	0.496	0.462	0.439
	Plug	0.119	0.130	0.106	0.099	0.149	0.002	0.001	0.002	0.001	0.002	0.412	0.431	0.510	0.427	0.449
	Womersley	0.142	0.138	0.141	0.109	0.155	0.003	0.002	0.003	0.005	0.002	0.401	0.419	0.485	0.418	0.449
7A	Parabolic	0.120	0.129	0.110	0.095	0.127	0.003	0.002	0.003	0.004	0.002	0.297	0.309	0.371	0.317	0.331
	Plug	0.084	0.101	0.083	0.065	0.098	0.002	0.001	0.002	0.005	0.001	0.300	0.308	0.367	0.323	0.330
	Womersley	0.098	0.108	0.092	0.083	0.110	0.003	0.001	0.002	0.003	0.002	0.303	0.310	0.366	0.334	0.335
14B	Parabolic	0.113	0.129	0.102	0.078	0.143	0.003	0.003	0.004	0.005	0.001	0.256	0.261	0.344	0.286	0.293
	Plug	0.298	0.308	0.271	0.259	0.321	0.004	0.004	0.007	0.007	0.002	0.262	0.266	0.349	0.289	0.301
	Womersley	0.313	0.319	0.274	0.256	0.336	0.005	0.005	0.005	0.007	0.002	0.261	0.266	0.347	0.288	0.302
16A	Parabolic	0.088	0.099	0.082	0.067	0.105	0.001	0.001	0.001	0.002	0.001	0.299	0.300	0.370	0.322	0.330
	Plug	0.105	0.117	0.098	0.091	0.116	0.001	0.001	0.004	0.005	0.001	0.296	0.301	0.366	0.318	0.328
	Womersley	0.115	0.126	0.105	0.089	0.131	0.007	0.001	0.001	0.001	0.001	0.292	0.301	0.359	0.305	0.333
31A	Parabolic	0.123	0.132	0.112	0.086	0.139	0.000	0.000	0.001	0.001	0.000	0.386	0.392	0.494	0.423	0.410
	Plug	0.140	0.149	0.132	0.110	0.162	0.001	0.001	0.001	0.001	0.001	0.381	0.391	0.469	0.412	0.418
	Womersley	0.146	0.161	0.138	0.108	0.164	0.001	0.000	0.000	0.000	0.000	0.390	0.395	0.478	0.418	0.421
41B	Parabolic	0.121	0.128	0.118	0.093	0.140	0.002	0.002	0.002	0.004	0.002	0.420	0.425	0.497	0.460	0.443
	Plug	0.130	0.133	0.122	0.106	0.139	0.002	0.002	0.002	0.002	0.002	0.431	0.428	0.511	0.464	0.443
	Womersley	0.135	0.138	0.125	0.106	0.144	0.003	0.003	0.003	0.004	0.002	0.432	0.428	0.515	0.459	0.444
63A	Parabolic	0.132	0.147	0.120	0.096	0.161	0.001	0.005	0.001	0.001	0.000	0.308	0.316	0.366	0.318	0.349
	Plug	0.088	0.104	0.080	0.061	0.112	0.001	0.000	0.001	0.001	0.000	0.315	0.323	0.378	0.326	0.351
	Womersley	0.132	0.141	0.120	0.114	0.150	0.001	0.000	0.001	0.001	0.000	0.314	0.322	0.375	0.324	0.350

## Data Availability

Statistical investigations are fully reproducible as all produced and analyzed data are available upon request; replications of outlier- and heteroscedasticity-robust statistical outcomes can be performed with R package WRS2.

## Nomenclature

**U**	fluid velocity
P	fluid pressure
ρ	fluid density
τ	stress tensor
D	rate-of-deformation tensor
$\dot{\gamma }$	shear rate
μ	dynamic viscosity
IVD	Inlet Velocity Distribution
CY	Carreau–Yasuda
Cs	Casson
HB	Herschel–Bulkley
N	Newtonian
P	Power law
WSS	wall shear stress
TAWSS	time average wall shear stress
OSI	oscillatory shear index
RRT	relative residence time
f_A_	flow asymmetry
f_D_	flow dispersion
$V_{\max}^{15\%}$	top 15% peak systolic velocity
ANOVA	Analysis of variance
CI	Confidence Interval
p	order of convergence
r	grid refinement ratio
GCI	Grid Convergence Index
$F_{s}$	safety factor
